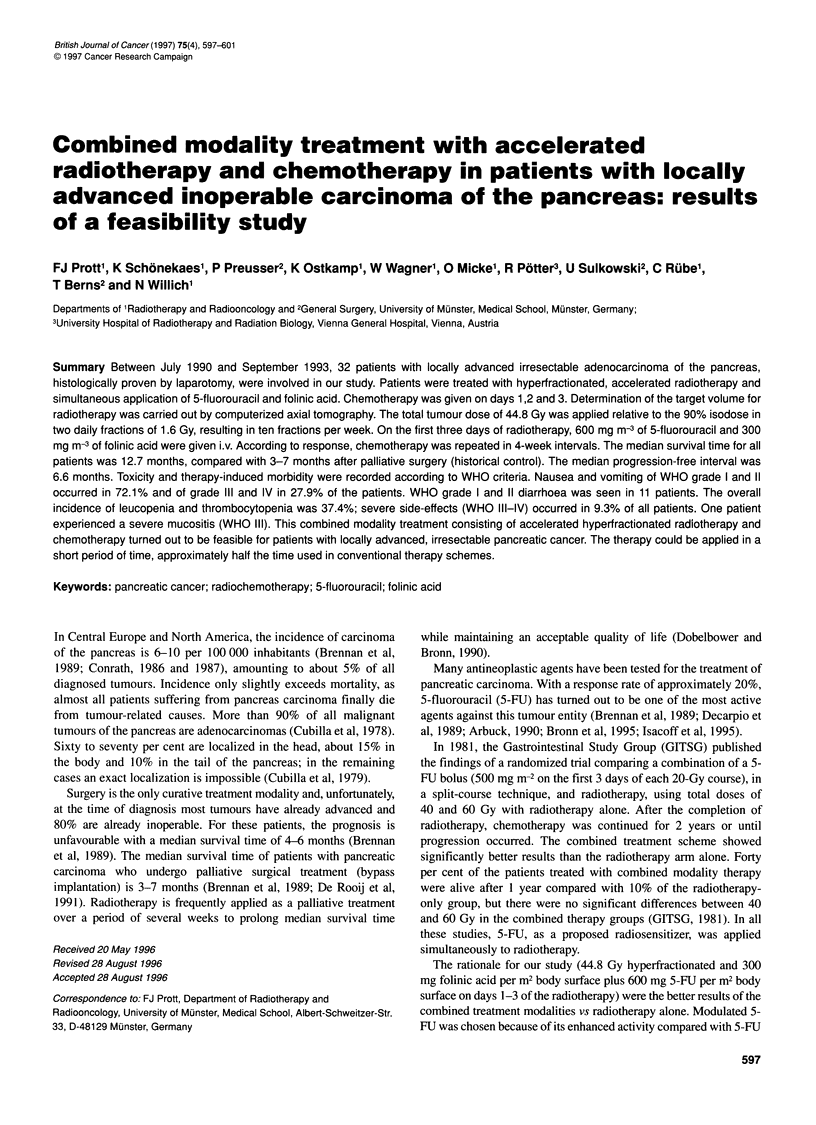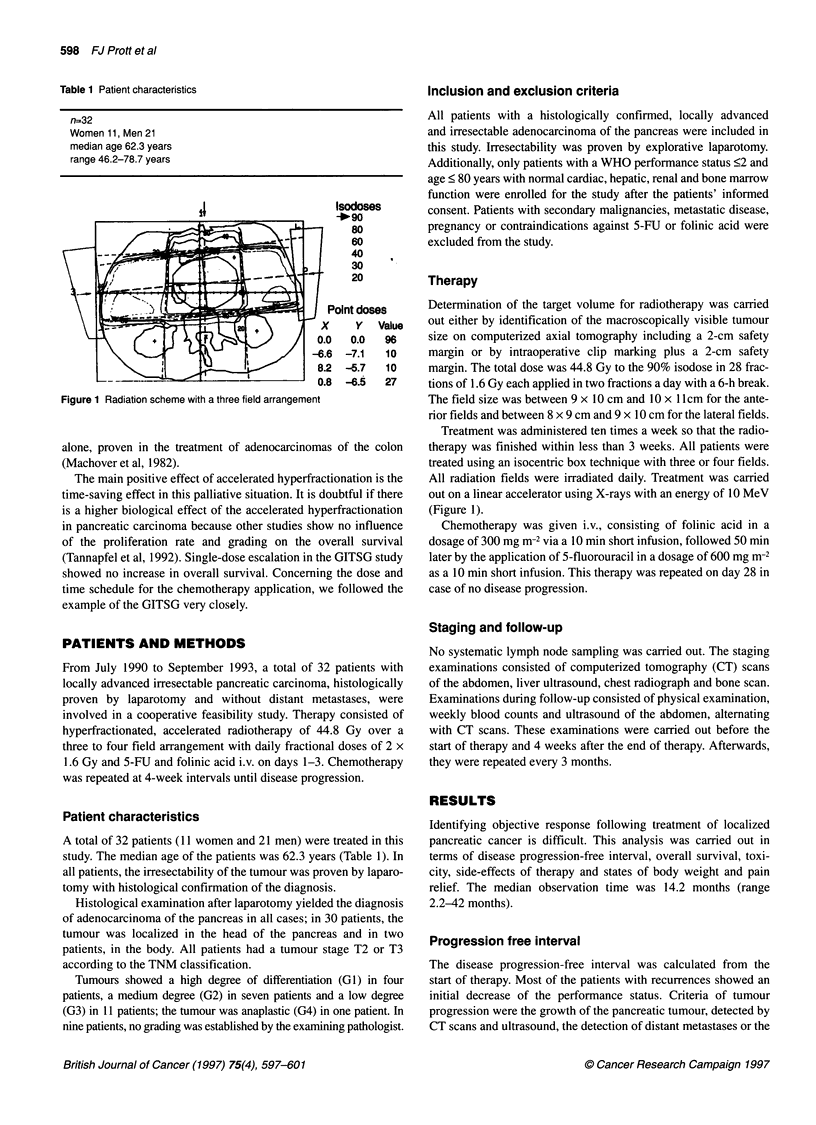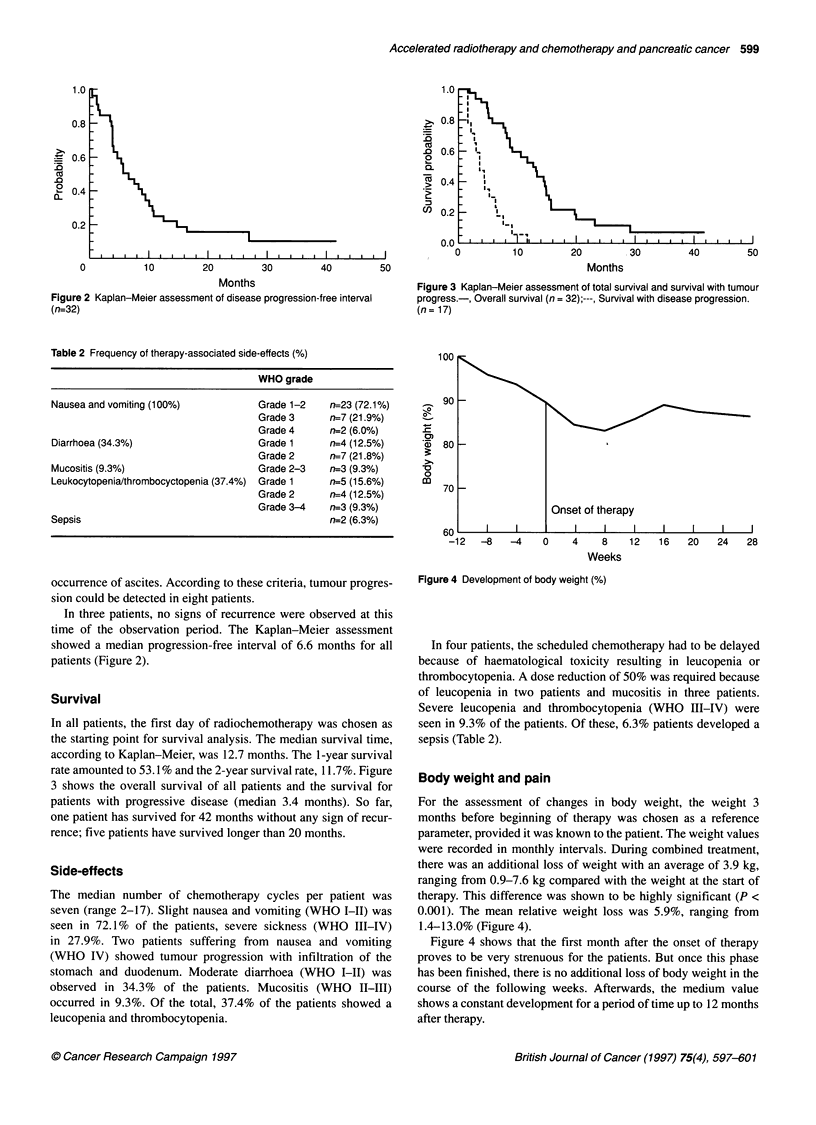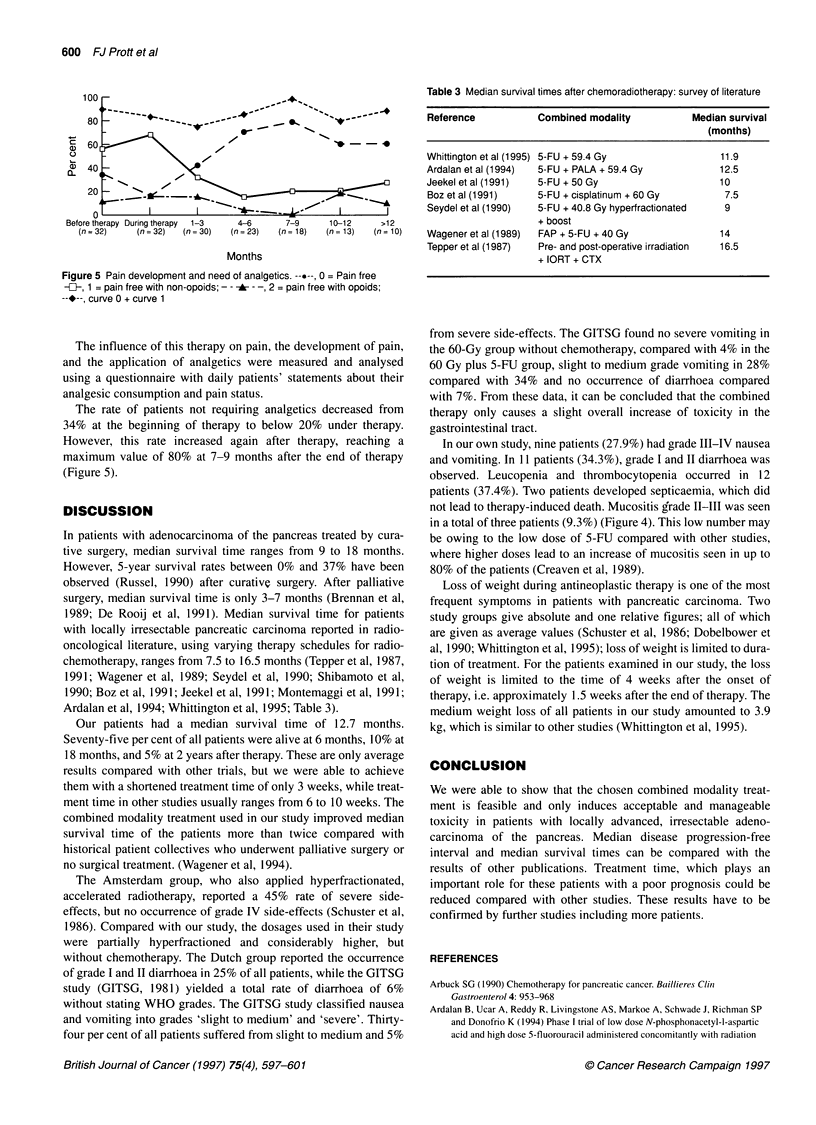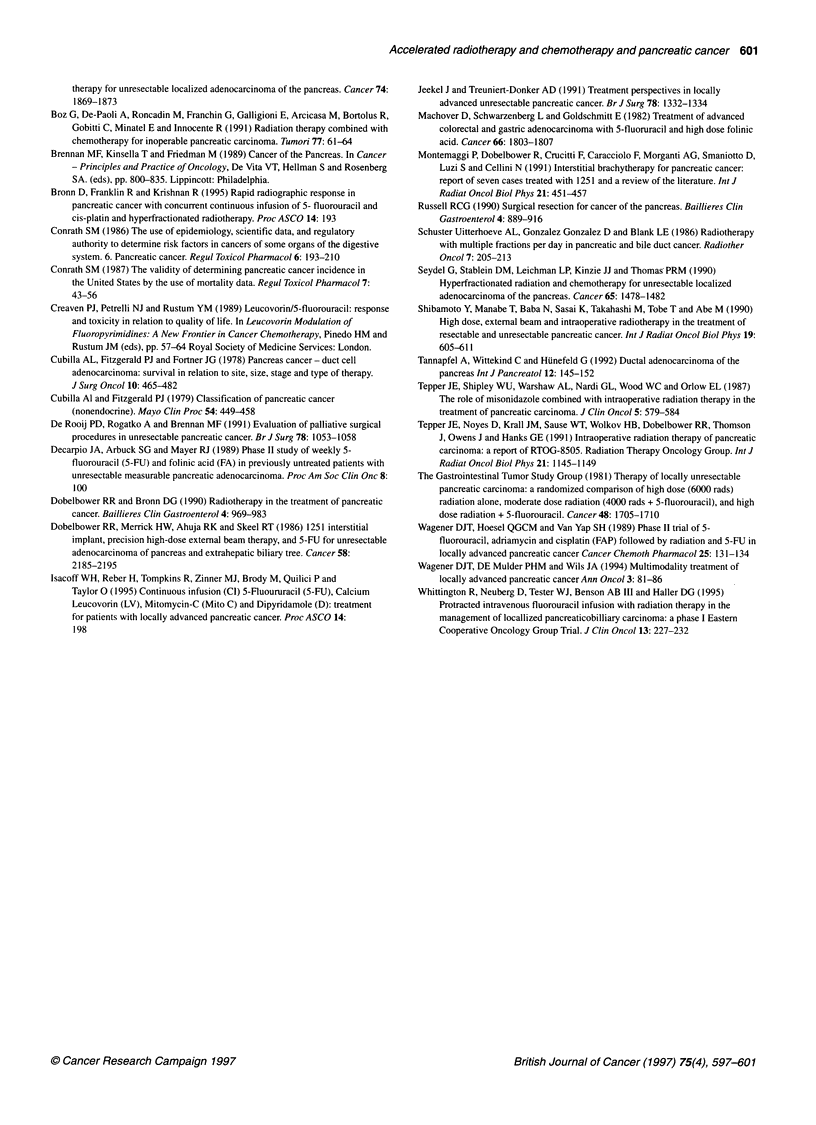# Combined modality treatment with accelerated radiotherapy and chemotherapy in patients with locally advanced inoperable carcinoma of the pancreas: results of a feasibility study.

**DOI:** 10.1038/bjc.1997.104

**Published:** 1997

**Authors:** F. J. Prott, K. Schönekaes, P. Preusser, K. Ostkamp, W. Wagner, O. Micke, R. Pötter, U. Sulkowski, C. Rübe, T. Berns, N. Willich

**Affiliations:** Department of Radiotherapy, University of Münster, Medical School, Germany.

## Abstract

Between July 1990 and September 1993, 32 patients with locally advanced irresectable adenocarcinoma of the pancreas, histologically proven by laparotomy, were involved in our study. Patients were treated with hyperfractionated, accelerated radiotherapy and simultaneous application of 5-fluorouracil and folinic acid. Chemotherapy was given on days 1,2 and 3. Determination of the target volume for radiotherapy was carried out by computerized axial tomography. The total tumour dose of 44.8 Gy was applied relative to the 90% isodose in two daily fractions of 1.6 Gy, resulting in ten fractions per week. On the first three days of radiotherapy, 600 mg m-3 of 5-fluorouracil and 300 mg m-3 of folinic acid were given i.v. According to response, chemotherapy was repeated in 4-week intervals. The median survival time for all patients was 12.7 months, compared with 3-7 months after palliative surgery (historical control). The median progression-free interval was 6.6 months. Toxicity and therapy-induced morbidity were recorded according to WHO criteria. Nausea and vomiting of WHO grade I and II occurred in 72.1% and of grade III and IV in 27.9% of the patients. WHO grade I and II diarrhoea was seen in 11 patients. The overall incidence of leucopenia and thrombocytopenia was 37.4%; severe side-effects (WHO III-IV) occurred in 9.3% of all patients. One patient experienced a severe mucositis (WHO III). This combined modality treatment consisting of accelerated hyperfractionated radiotherapy and chemotherapy turned out to be feasible for patients with locally advanced, irresectable pancreatic cancer. The therapy could be applied in a short period of time, approximately half the time used in conventional therapy schemes.


British Journal of Cancer (1997) 75(4), 597–601
© 1997 Cancer Research Campaign

### Combined modality treatment with accelerated radiotherapy and chemotherapy in patients with locally advanced inoperable carcinoma of the pancreas: results of a feasibility study

FJ Prott[1], K Schönekaes[1], P Preusser[2], K Ostkamp[1], W Wagner[1], O Micke[1], R Pötter[3], U Sulkowski[2], C Rübe[1], T Berns[2] and N Willich[1]

Departments of [1]Radiotherapy and Radiooncology and [2]General Surgery, University of Münster, Medical School, Münster, Germany; [3]University Hospital of Radiotherapy and Radiation Biology, Vienna General Hospital, Vienna, Austria

**Summary** Between July 1990 and September 1993, 32 patients with locally advanced irresectable adenocarcinoma of the pancreas, histologically proven by laparotomy, were involved in our study. Patients were treated with hyperfractionated, accelerated radiotherapy and simultaneous application of 5-fluorouracil and folinic acid. Chemotherapy was given on days 1,2 and 3. Determination of the target volume for radiotherapy was carried out by computerized axial tomography. The total tumour dose of 44.8 Gy was applied relative to the 90% isodose in two daily fractions of 1.6 Gy, resulting in ten fractions per week. On the first three days of radiotherapy, 600 mg $m^{-3}$ of 5-fluorouracil and 300 mg $m^{-3}$ of folinic acid were given i.v. According to response, chemotherapy was repeated in 4-week intervals. The median survival time for all patients was 12.7 months, compared with 3–7 months after palliative surgery (historical control). The median progression-free interval was 6.6 months. Toxicity and therapy-induced morbidity were recorded according to WHO criteria. Nausea and vomiting of WHO grade I and II occurred in 72.1% and of grade III and IV in 27.9% of the patients. WHO grade I and II diarrhoea was seen in 11 patients. The overall incidence of leucopenia and thrombocytopenia was 37.4%; severe side-effects (WHO III–IV) occurred in 9.3% of all patients. One patient experienced a severe mucositis (WHO III). This combined modality treatment consisting of accelerated hyperfractionated radiotherapy and chemotherapy turned out to be feasible for patients with locally advanced, irresectable pancreatic cancer. The therapy could be applied in a short period of time, approximately half the time used in conventional therapy schemes.

**Keywords:** pancreatic cancer; radiochemotherapy; 5-fluorouracil; folinic acid

In Central Europe and North America, the incidence of carcinoma of the pancreas is 6–10 per 100 000 inhabitants (Brennan et al, 1989; Conrath, 1986 and 1987), amounting to about 5% of all diagnosed tumours. Incidence only slightly exceeds mortality, as almost all patients suffering from pancreas carcinoma finally die from tumour-related causes. More than 90% of all malignant tumours of the pancreas are adenocarcinomas (Cubilla et al, 1978). Sixty to seventy per cent are localized in the head, about 15% in the body and 10% in the tail of the pancreas; in the remaining cases an exact localization is impossible (Cubilla et al, 1979).

Surgery is the only curative treatment modality and, unfortunately, at the time of diagnosis most tumours have already advanced and 80% are already inoperable. For these patients, the prognosis is unfavourable with a median survival time of 4–6 months (Brennan et al, 1989). The median survival time of patients with pancreatic carcinoma who undergo palliative surgical treatment (bypass implantation) is 3–7 months (Brennan et al, 1989; De Rooij et al, 1991). Radiotherapy is frequently applied as a palliative treatment over a period of several weeks to prolong median survival time while maintaining an acceptable quality of life (Dobelbower and Bronn, 1990).

Many antineoplastic agents have been tested for the treatment of pancreatic carcinoma. With a response rate of approximately 20%, 5-fluorouracil (5-FU) has turned out to be one of the most active agents against this tumour entity (Brennan et al, 1989; Decarpio et al, 1989; Arbuck, 1990; Bronn et al, 1995; Isacoff et al, 1995).

In 1981, the Gastrointestinal Study Group (GITSG) published the findings of a randomized trial comparing a combination of a 5-FU bolus (500 mg $m^{-2}$ on the first 3 days of each 20-Gy course), in a split-course technique, and radiotherapy, using total doses of 40 and 60 Gy with radiotherapy alone. After the completion of radiotherapy, chemotherapy was continued for 2 years or until progression occurred. The combined treatment scheme showed significantly better results than the radiotherapy arm alone. Forty per cent of the patients treated with combined modality therapy were alive after 1 year compared with 10% of the radiotherapy-only group, but there were no significant differences between 40 and 60 Gy in the combined therapy groups (GITSG, 1981). In all these studies, 5-FU, as a proposed radiosensitizer, was applied simultaneously to radiotherapy.

The rationale for our study (44.8 Gy hyperfractionated and 300 mg folinic acid per $m^2$ body surface plus 600 mg 5-FU per $m^2$ body surface on days 1–3 of the radiotherapy) were the better results of the combined treatment modalities *vs* radiotherapy alone. Modulated 5-FU was chosen because of its enhanced activity compared with 5-FU

*Received 20 May 1996*
*Revised 28 August 1996*
*Accepted 28 August 1996*

*Correspondence to:* FJ Prott, Department of Radiotherapy and Radiooncology, University of Münster, Medical School, Albert-Schweitzer-Str. 33, D-48129 Münster, Germany

**Table 1** Patient characteristics

| |
|---|
| *n*=32 |
| Women 11, Men 21 |
| median age 62.3 years |
| range 46.2–78.7 years |

**Figure 1** Radiation scheme with a three field arrangement alone, proven in the treatment of adenocarcinomas of the colon (Machover et al, 1982).

The main positive effect of accelerated hyperfractionation is the time-saving effect in this palliative situation. It is doubtful if there is a higher biological effect of the accelerated hyperfractionation in pancreatic carcinoma because other studies show no influence of the proliferation rate and grading on the overall survival (Tannapfel et al, 1992). Single-dose escalation in the GITSG study showed no increase in overall survival. Concerning the dose and time schedule for the chemotherapy application, we followed the example of the GITSG very closely.

### PATIENTS AND METHODS

From July 1990 to September 1993, a total of 32 patients with locally advanced irresectable pancreatic carcinoma, histologically proven by laparotomy and without distant metastases, were involved in a cooperative feasibility study. Therapy consisted of hyperfractionated, accelerated radiotherapy of 44.8 Gy over a three to four field arrangement with daily fractional doses of 2 × 1.6 Gy and 5-FU and folinic acid i.v. on days 1–3. Chemotherapy was repeated at 4-week intervals until disease progression.

### Patient characteristics

A total of 32 patients (11 women and 21 men) were treated in this study. The median age of the patients was 62.3 years (Table 1). In all patients, the irresectability of the tumour was proven by laparotomy with histological confirmation of the diagnosis.

Histological examination after laparotomy yielded the diagnosis of adenocarcinoma of the pancreas in all cases; in 30 patients, the tumour was localized in the head of the pancreas and in two patients, in the body. All patients had a tumour stage T2 or T3 according to the TNM classification.

Tumours showed a high degree of differentiation (G1) in four patients, a medium degree (G2) in seven patients and a low degree (G3) in 11 patients; the tumour was anaplastic (G4) in one patient. In nine patients, no grading was established by the examining pathologist.

### Inclusion and exclusion criteria

All patients with a histologically confirmed, locally advanced and irresectable adenocarcinoma of the pancreas were included in this study. Irresectability was proven by explorative laparotomy. Additionally, only patients with a WHO performance status ≤2 and age ≤ 80 years with normal cardiac, hepatic, renal and bone marrow function were enrolled for the study after the patients' informed consent. Patients with secondary malignancies, metastatic disease, pregnancy or contraindications against 5-FU or folinic acid were excluded from the study.

### Therapy

Determination of the target volume for radiotherapy was carried out either by identification of the macroscopically visible tumour size on computerized axial tomography including a 2-cm safety margin or by intraoperative clip marking plus a 2-cm safety margin. The total dose was 44.8 Gy to the 90% isodose in 28 fractions of 1.6 Gy each applied in two fractions a day with a 6-h break. The field size was between 9 × 10 cm and 10 × 11cm for the anterior fields and between 8 × 9 cm and 9 × 10 cm for the lateral fields.

Treatment was administered ten times a week so that the radiotherapy was finished within less than 3 weeks. All patients were treated using an isocentric box technique with three or four fields. All radiation fields were irradiated daily. Treatment was carried out on a linear accelerator using X-rays with an energy of 10 MeV (Figure 1).

Chemotherapy was given i.v., consisting of folinic acid in a dosage of 300 mg $m^{-2}$ via a 10 min short infusion, followed 50 min later by the application of 5-fluorouracil in a dosage of 600 mg $m^{-2}$ as a 10 min short infusion. This therapy was repeated on day 28 in case of no disease progression.

### Staging and follow-up

No systematic lymph node sampling was carried out. The staging examinations consisted of computerized tomography (CT) scans of the abdomen, liver ultrasound, chest radiograph and bone scan. Examinations during follow-up consisted of physical examination, weekly blood counts and ultrasound of the abdomen, alternating with CT scans. These examinations were carried out before the start of therapy and 4 weeks after the end of therapy. Afterwards, they were repeated every 3 months.

### RESULTS

Identifying objective response following treatment of localized pancreatic cancer is difficult. This analysis was carried out in terms of disease progression-free interval, overall survival, toxicity, side-effects of therapy and states of body weight and pain relief. The median observation time was 14.2 months (range 2.2–42 months).

### Progression free interval

The disease progression-free interval was calculated from the start of therapy. Most of the patients with recurrences showed an initial decrease of the performance status. Criteria of tumour progression were the growth of the pancreatic tumour, detected by CT scans and ultrasound, the detection of distant metastases or the

© Cancer Research Campaign 1997

**Figure 2** Kaplan–Meier assessment of disease progression-free interval ($n$=32)

**Figure 3** Kaplan–Meier assessment of total survival and survival with tumour progress.—, Overall survival ($n$ = 32);---, Survival with disease progression. ($n$ = 17)

**Table 2** Frequency of therapy-associated side-effects (%)

| | WHO grade | |
|---|---|---|
| Nausea and vomiting (100%) | Grade 1–2 | $n$=23 (72.1%) |
| | Grade 3 | $n$=7 (21.9%) |
| | Grade 4 | $n$=2 (6.0%) |
| Diarrhoea (34.3%) | Grade 1 | $n$=4 (12.5%) |
| | Grade 2 | $n$=7 (21.8%) |
| Mucositis (9.3%) | Grade 2–3 | $n$=3 (9.3%) |
| Leukocytopenia/thrombocyctopenia (37.4%) | Grade 1 | $n$=5 (15.6%) |
| | Grade 2 | $n$=4 (12.5%) |
| | Grade 3–4 | $n$=3 (9.3%) |
| Sepsis | | $n$=2 (6.3%) |

**Figure 4** Development of body weight (%)

occurrence of ascites. According to these criteria, tumour progression could be detected in eight patients.

In three patients, no signs of recurrence were observed at this time of the observation period. The Kaplan–Meier assessment showed a median progression-free interval of 6.6 months for all patients (Figure 2).

### Survival

In all patients, the first day of radiochemotherapy was chosen as the starting point for survival analysis. The median survival time, according to Kaplan–Meier, was 12.7 months. The 1-year survival rate amounted to 53.1% and the 2-year survival rate, 11.7%. Figure 3 shows the overall survival of all patients and the survival for patients with progressive disease (median 3.4 months). So far, one patient has survived for 42 months without any sign of recurrence; five patients have survived longer than 20 months.

### Side-effects

The median number of chemotherapy cycles per patient was seven (range 2–17). Slight nausea and vomiting (WHO I–II) was seen in 72.1% of the patients, severe sickness (WHO III–IV) in 27.9%. Two patients suffering from nausea and vomiting (WHO IV) showed tumour progression with infiltration of the stomach and duodenum. Moderate diarrhoea (WHO I–II) was observed in 34.3% of the patients. Mucositis (WHO II–III) occurred in 9.3%. Of the total, 37.4% of the patients showed a leucopenia and thrombocytopenia.

In four patients, the scheduled chemotherapy had to be delayed because of haematological toxicity resulting in leucopenia or thrombocytopenia. A dose reduction of 50% was required because of leucopenia in two patients and mucositis in three patients. Severe leucopenia and thrombocytopenia (WHO III–IV) were seen in 9.3% of the patients. Of these, 6.3% patients developed a sepsis (Table 2).

### Body weight and pain

For the assessment of changes in body weight, the weight 3 months before beginning of therapy was chosen as a reference parameter, provided it was known to the patient. The weight values were recorded in monthly intervals. During combined treatment, there was an additional loss of weight with an average of 3.9 kg, ranging from 0.9–7.6 kg compared with the weight at the start of therapy. This difference was shown to be highly significant ($P$ < 0.001). The mean relative weight loss was 5.9%, ranging from 1.4–13.0% (Figure 4).

Figure 4 shows that the first month after the onset of therapy proves to be very strenuous for the patients. But once this phase has been finished, there is no additional loss of body weight in the course of the following weeks. Afterwards, the medium value shows a constant development for a period of time up to 12 months after therapy.

© Cancer Research Campaign 1997

**Figure 5** Pain development and need of analgetics. --•--, 0 = Pain free -□-, 1 = pain free with non-opoids; – - ▲ - –, 2 = pain free with opoids; --♦--, curve 0 + curve 1

**Table 3** Median survival times after chemoradiotherapy: survey of literature

| Reference | Combined modality | Median survival (months) |
|---|---|---|
| Whittington et al (1995) | 5-FU + 59.4 Gy | 11.9 |
| Ardalan et al (1994) | 5-FU + PALA + 59.4 Gy | 12.5 |
| Jeekel et al (1991) | 5-FU + 50 Gy | 10 |
| Boz et al (1991) | 5-FU + cisplatinum + 60 Gy | 7.5 |
| Seydel et al (1990) | 5-FU + 40.8 Gy hyperfractionated + boost | 9 |
| Wagener et al (1989) | FAP + 5-FU + 40 Gy | 14 |
| Tepper et al (1987) | Pre- and post-operative irradiation + IORT + CTX | 16.5 |

The influence of this therapy on pain, the development of pain, and the application of analgetics were measured and analysed using a questionnaire with daily patients' statements about their analgesic consumption and pain status.

The rate of patients not requiring analgetics decreased from 34% at the beginning of therapy to below 20% under therapy. However, this rate increased again after therapy, reaching a maximum value of 80% at 7–9 months after the end of therapy (Figure 5).

### DISCUSSION

In patients with adenocarcinoma of the pancreas treated by curative surgery, median survival time ranges from 9 to 18 months. However, 5-year survival rates between 0% and 37% have been observed (Russel, 1990) after curative surgery. After palliative surgery, median survival time is only 3–7 months (Brennan et al, 1989; De Rooij et al, 1991). Median survival time for patients with locally irresectable pancreatic carcinoma reported in radio-oncological literature, using varying therapy schedules for radiochemotherapy, ranges from 7.5 to 16.5 months (Tepper et al, 1987, 1991; Wagener et al, 1989; Seydel et al, 1990; Shibamoto et al, 1990; Boz et al, 1991; Jeekel et al, 1991; Montemaggi et al, 1991; Ardalan et al, 1994; Whittington et al, 1995; Table 3).

Our patients had a median survival time of 12.7 months. Seventy-five per cent of all patients were alive at 6 months, 10% at 18 months, and 5% at 2 years after therapy. These are only average results compared with other trials, but we were able to achieve them with a shortened treatment time of only 3 weeks, while treatment time in other studies usually ranges from 6 to 10 weeks. The combined modality treatment used in our study improved median survival time of the patients more than twice compared with historical patient collectives who underwent palliative surgery or no surgical treatment. (Wagener et al, 1994).

The Amsterdam group, who also applied hyperfractionated, accelerated radiotherapy, reported a 45% rate of severe side-effects, but no occurrence of grade IV side-effects (Schuster et al, 1986). Compared with our study, the dosages used in their study were partially hyperfractioned and considerably higher, but without chemotherapy. The Dutch group reported the occurrence of grade I and II diarrhoea in 25% of all patients, while the GITSG study (GITSG, 1981) yielded a total rate of diarrhoea of 6% without stating WHO grades. The GITSG study classified nausea and vomiting into grades 'slight to medium' and 'severe'. Thirty-four per cent of all patients suffered from slight to medium and 5% from severe side-effects. The GITSG found no severe vomiting in the 60-Gy group without chemotherapy, compared with 4% in the 60 Gy plus 5-FU group, slight to medium grade vomiting in 28% compared with 34% and no occurrence of diarrhoea compared with 7%. From these data, it can be concluded that the combined therapy only causes a slight overall increase of toxicity in the gastrointestinal tract.

In our own study, nine patients (27.9%) had grade III–IV nausea and vomiting. In 11 patients (34.3%), grade I and II diarrhoea was observed. Leucopenia and thrombocytopenia occurred in 12 patients (37.4%). Two patients developed septicaemia, which did not lead to therapy-induced death. Mucositis grade II–III was seen in a total of three patients (9.3%) (Figure 4). This low number may be owing to the low dose of 5-FU compared with other studies, where higher doses lead to an increase of mucositis seen in up to 80% of the patients (Creaven et al, 1989).

Loss of weight during antineoplastic therapy is one of the most frequent symptoms in patients with pancreatic carcinoma. Two study groups give absolute and one relative figures; all of which are given as average values (Schuster et al, 1986; Dobelbower et al, 1990; Whittington et al, 1995); loss of weight is limited to duration of treatment. For the patients examined in our study, the loss of weight is limited to the time of 4 weeks after the onset of therapy, i.e. approximately 1.5 weeks after the end of therapy. The medium weight loss of all patients in our study amounted to 3.9 kg, which is similar to other studies (Whittington et al, 1995).

### CONCLUSION

We were able to show that the chosen combined modality treatment is feasible and only induces acceptable and manageable toxicity in patients with locally advanced, irresectable adenocarcinoma of the pancreas. Median disease progression-free interval and median survival times can be compared with the results of other publications. Treatment time, which plays an important role for these patients with a poor prognosis could be reduced compared with other studies. These results have to be confirmed by further studies including more patients.

### REFERENCES

Arbuck SG (1990) Chemotherapy for pancreatic cancer. *Baillieres Clin Gastroenterol* 4: 953–968

Ardalan B, Ucar A, Reddy R, Livingstone AS, Markoe A, Schwade J, Richman SP and Donofrio K (1994) Phase I trial of low dose *N*-phosphonacetyl-l-aspartic acid and high dose 5-fluorouracil administered concomitantly with radiation

© Cancer Research Campaign 1997

therapy for unresectable localized adenocarcinoma of the pancreas. *Cancer* **74**: 1869–1873

Boz G, De-Paoli A, Roncadin M, Franchin G, Galligioni E, Arcicasa M, Bortolus R, Gobitti C, Minatel E and Innocente R (1991) Radiation therapy combined with chemotherapy for inoperable pancreatic carcinoma. *Tumori* **77**: 61–64

Brennan MF, Kinsella T and Friedman M (1989) Cancer of the Pancreas. In *Cancer – Principles and Practice of Oncology*, De Vita VT, Hellman S and Rosenberg SA. (eds), pp. 800–835. Lippincott: Philadelphia.

Bronn D, Franklin R and Krishnan R (1995) Rapid radiographic response in pancreatic cancer with concurrent continuous infusion of 5- fluorouracil and cis-platin and hyperfractionated radiotherapy. *Proc ASCO* **14**: 193

Conrath SM (1986) The use of epidemiology, scientific data, and regulatory authority to determine risk factors in cancers of some organs of the digestive system. 6. Pancreatic cancer. *Regul Toxicol Pharmacol* **6**: 193–210

Conrath SM (1987) The validity of determining pancreatic cancer incidence in the United States by the use of mortality data. *Regul Toxicol Pharmacol* **7**: 43-56

Creaven PJ, Petrelli NJ and Rustum YM (1989) Leucovorin/5-fluorouracil: response and toxicity in relation to quality of life. In *Leucovorin Modulation of Fluoropyrimidines: A New Frontier in Cancer Chemotherapy*, Pinedo HM and Rustum JM (eds), pp. 57–64 Royal Society of Medicine Services: London.

Cubilla AL, Fitzgerald PJ and Fortner JG (1978) Pancreas cancer – duct cell adenocarcinoma: survival in relation to site, size, stage and type of therapy. *J Surg Oncol* **10**: 465–482

Cubilla Al and Fitzgerald PJ (1979) Classification of pancreatic cancer (nonendocrine). *Mayo Clin Proc* **54**: 449–458

De Rooij PD, Rogatko A and Brennan MF (1991) Evaluation of palliative surgical procedures in unresectable pancreatic cancer. *Br J Surg* **78**: 1053–1058

Decarpio JA, Arbuck SG and Mayer RJ (1989) Phase II study of weekly 5-fluorouracil (5-FU) and folinic acid (FA) in previously untreated patients with unresectable measurable pancreatic adenocarcinoma. *Proc Am Soc Clin Onc* **8**: 100

Dobelbower RR and Bronn DG (1990) Radiotherapy in the treatment of pancreatic cancer. *Baillieres Clin Gastroenterol* **4**: 969–983

Dobelbower RR, Merrick HW, Ahuja RK and Skeel RT (1986) 1251 interstitial implant, precision high-dose external beam therapy, and 5-FU for unresectable adenocarcinoma of pancreas and extrahepatic biliary tree. *Cancer* **58**: 2185–2195

Isacoff WH, Reber H, Tompkins R, Zinner MJ, Brody M, Quilici P and Taylor O (1995) Continuous infusion (CI) 5-Fluoururacil (5-FU), Calcium Leucovorin (LV), Mitomycin-C (Mito C) and Dipyridamole (D): treatment for patients with locally advanced pancreatic cancer. *Proc ASCO* **14**: 198

Jeekel J and Treuniert-Donker AD (1991) Treatment perspectives in locally advanced unresectable pancreatic cancer. *Br J Surg* **78**: 1332–1334

Machover D, Schwarzenberg L and Goldschmitt E (1982) Treatment of advanced colorectal and gastric adenocarcinoma with 5-fluoruracil and high dose folinic acid. *Cancer* **66**: 1803–1807

Montemaggi P, Dobelbower R, Crucitti F, Caracciolo F, Morganti AG, Smaniotto D, Luzi S and Cellini N (1991) Interstitial brachytherapy for pancreatic cancer: report of seven cases treated with 1251 and a review of the literature. *Int J Radiat Oncol Biol Phys* **21**: 451–457

Russell RCG (1990) Surgical resection for cancer of the pancreas. *Baillieres Clin Gastroenterol* **4**: 889–916

Schuster Uitterhoeve AL, Gonzalez Gonzalez D and Blank LE (1986) Radiotherapy with multiple fractions per day in pancreatic and bile duct cancer. *Radiother Oncol* **7**: 205–213

Seydel G, Stablein DM, Leichman LP, Kinzie JJ and Thomas PRM (1990) Hyperfractionated radiation and chemotherapy for unresectable localized adenocarcinoma of the pancreas. *Cancer* **65**: 1478–1482

Shibamoto Y, Manabe T, Baba N, Sasai K, Takahashi M, Tobe T and Abe M (1990) High dose, external beam and intraoperative radiotherapy in the treatment of resectable and unresectable pancreatic cancer. *Int J Radiat Oncol Biol Phys* **19**: 605–611

Tannapfel A, Wittekind C and Hünefeld G (1992) Ductal adenocarcinoma of the pancreas *Int J Pancreatol* **12**: 145–152

Tepper JE, Shipley WU, Warshaw AL, Nardi GL, Wood WC and Orlow EL (1987) The role of misonidazole combined with intraoperative radiation therapy in the treatment of pancreatic carcinoma. *J Clin Oncol* **5**: 579–584

Tepper JE, Noyes D, Krall JM, Sause WT, Wolkov HB, Dobelbower RR, Thomson J, Owens J and Hanks GE (1991) Intraoperative radiation therapy of pancreatic carcinoma: a report of RTOG-8505. Radiation Therapy Oncology Group. *Int J Radiat Oncol Biol Phys* **21**: 1145–1149

The Gastrointestinal Tumor Study Group (1981) Therapy of locally unresectable pancreatic carcinoma: a randomized comparison of high dose (6000 rads) radiation alone, moderate dose radiation (4000 rads + 5-fluorouracil), and high dose radiation + 5-fluorouracil. *Cancer* **48**: 1705–1710

Wagener DJT, Hoesel QGCM and Van Yap SH (1989) Phase II trial of 5-fluorouracil, adriamycin and cisplatin (FAP) followed by radiation and 5-FU in locally advanced pancreatic cancer *Cancer Chemoth Pharmacol* **25**: 131–134

Wagener DJT, DE Mulder PHM and Wils JA (1994) Multimodality treatment of locally advanced pancreatic cancer *Ann Oncol* **3**: 81–86

Whittington R, Neuberg D, Tester WJ, Benson AB III and Haller DG (1995) Protracted intravenous fluorouracil infusion with radiation therapy in the management of locallized pancreaticobilliary carcinoma: a phase I Eastern Cooperative Oncology Group Trial. *J Clin Oncol* **13**: 227–232

© Cancer Research Campaign 1997